# A recurrence-tolerant strategy for epidermolysis bullosa-related pseudosyndactyly: A microscope-assisted minimally invasive approach

**DOI:** 10.1016/j.jpra.2026.04.029

**Published:** 2026-04-30

**Authors:** Kento Hosomi, Chihena Hansini Banda, Kohei Mitsui, Kohei Hashimoto, Kosuke Yamagata, Chizuki Kondo, Ryohei Ishiura, Mitsunaga Narushima

**Affiliations:** a Department of Plastic and Reconstructive Surgery, Mie University, 2-174 Edobashi Tsu city, Mie 514-8507, Japan; b Plastic and Reconstructive Surgery Unit, Department of Surgery, The University Teaching Hospital, Zambia

**Keywords:** Epidermolysis bullosa, Pseudosyndactyly, Microsurgery, Less invasive

## Abstract

Complete long-term prevention of recurrence in epidermolysis bullosa (EB)-related pseudosyndactyly remains challenging. We report a case applied a minimally invasive, recurrence-tolerant surgical strategy that allows repeatable intervention with reduced patient burden.

An 8-year-old boy with recessive dystrophic EB (RDEB) presented with advanced pseudosyndactyly. The patient and his mother expressed concerns regarding pain associated with treatment and postoperative care, as well as the need for repeated surgeries due to recurrence. Based on these concerns, a less invasive intervention was planned, focusing on selective and minimal release of scar tissue using microsurgical techniques.

Under general anesthesia, the epidermis and firm dermal layer were carefully incised using a microscope. Microsurgical perivascular dissection enabled additional release of contracture at the digital joints. A previously cryopreserved cultured epidermal autograft was applied to cover the wound surface, followed by non-adhesive dressing. The graft was secured with a tie-over dressing using a bandage, without pin fixation.

The patient was discharged on the day after surgery. Engraftment of the cultured epidermis was confirmed on postoperative day 4. Complete epithelialization was achieved within 3 weeks, after which rehabilitation was initiated. At 6 months postoperatively, no recurrent contracture was observed, and the improved range of motion was maintained.

Given the potential risk of recurrence in EB-related pseudosyndactyly, our surgical concept prioritizes repeatability in the event of contracture recurrence. The use of microsurgical techniques allowed real-time identification of the boundary between firm and soft dermal layers, achieving short-term functional improvement without recurrence.

## Introduction

In recessive dystrophic epidermolysis bullosa (RDEB), repeated blistering and ulceration lead to progressive scarring and the development of contractures. Especially in the hands, EB-related pseudosyndactyly impairs quality of life. Reconstructive procedures for digital separation have therefore long been performed and refined; however, the high long-term recurrence rate remains a major challenge.^1^, ^2^, ^3^, ^4^, ^5^

In this context, our primary objective was not complete prevention of recurrence, but rather the development of a minimally invasive surgical strategy that enables low-burden reintervention in the event of recurrence. In this report, we present a case of EB-related pseudosyndactyly treated using a microscope-assisted minimally invasive technique based on a recurrence-tolerant concept.

## Case presentation

**Figure 1:**
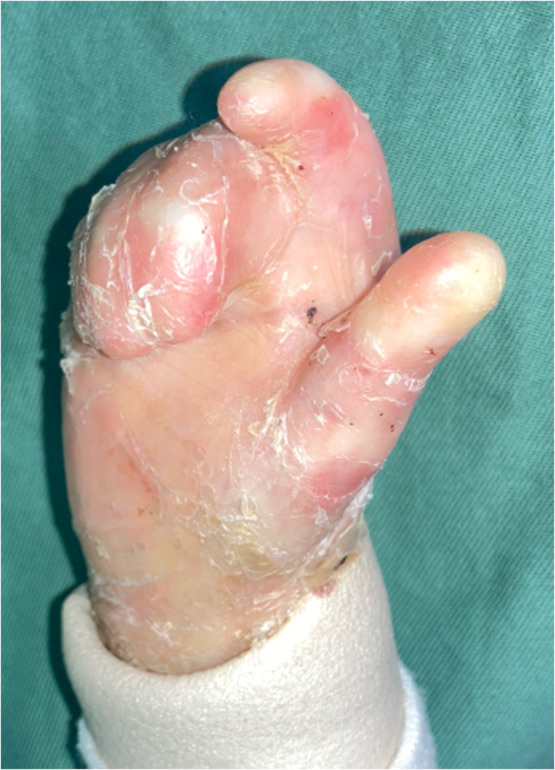
Preoperative view of the hand during extension.

An 8-year-old boy with RDEB presented with progressive pseudosyndactyly involving the index through little fingers, resulting in impairment of activities of daily living (Figure 1). The percentage of total active motion (%TAM) was 7.7% in the index finger and 3.9% in the middle through small fingers.

Based on concerns expressed by the patient and his mother, a surgical strategy prioritizing minimal invasiveness and repeatability was selected. The patient had previously undergone a cultured epidermal autograft (CEA; JACE®, Japan Tissue Engineering Co., Ltd., Aichi, Japan) at the age of two, which resulted in severe postoperative pain due to repeated dressing changes during hospitalization. Additionally, concerns were raised that the high long-term recurrence rate could necessitate repeated interventions associated with significant pain. Based on these considerations, a less invasive approach focused on the selective and minimal release of scar tissue using microsurgical techniques was adopted.

## Surgical procedure (Movie 1)

**Figure 2:**
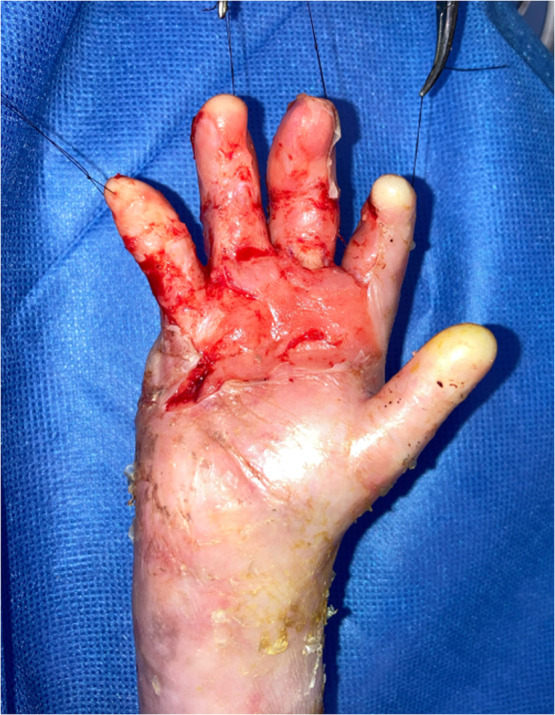
Immediate postoperative view after microscope-assisted digital separation.

**Figure 3:**
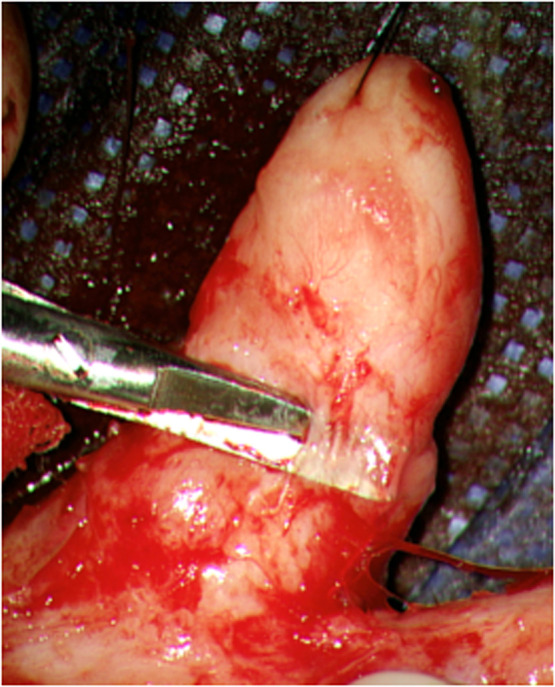
Microscopic assistance enables clear visualization of the layered structure within the thick dermis.

Under general anesthesia, the epidermis and firm dermis were carefully incised using a microscope (Figure 2). The border line of combined digits was easily detected by incision of epidermis, and separation of these borders was effectively achieved by blunt dissection. To prevent epidermal peeling, traction sutures were placed at the fingertip and gently applied during dissection. For further contracture release, microsurgical dissection enabled clear identification of the layer border between firm dermis and soft dermis (Figure 3). At the digital joints, full-thickness dermal incision was required to achieve adequate release of contracture, resulting in partial exposure of the proper palmar digital arteries. Microsurgical perivascular dissection allowed safe and controlled release of the scar contracture.

To avoid donor-site morbidity, the defects were covered with a CEA using a previously cryopreserved specimen. To maintain digital separation after finger extension, a non-adhesive dressing material (SI-Mesh®, ALCARE Co., Ltd., Japan) was applied to the wound surface and inserted into the web spaces. The graft was secured with a tie-over dressing using a bandage, without pin fixation.

## Postoperative care

**Figure 4:**
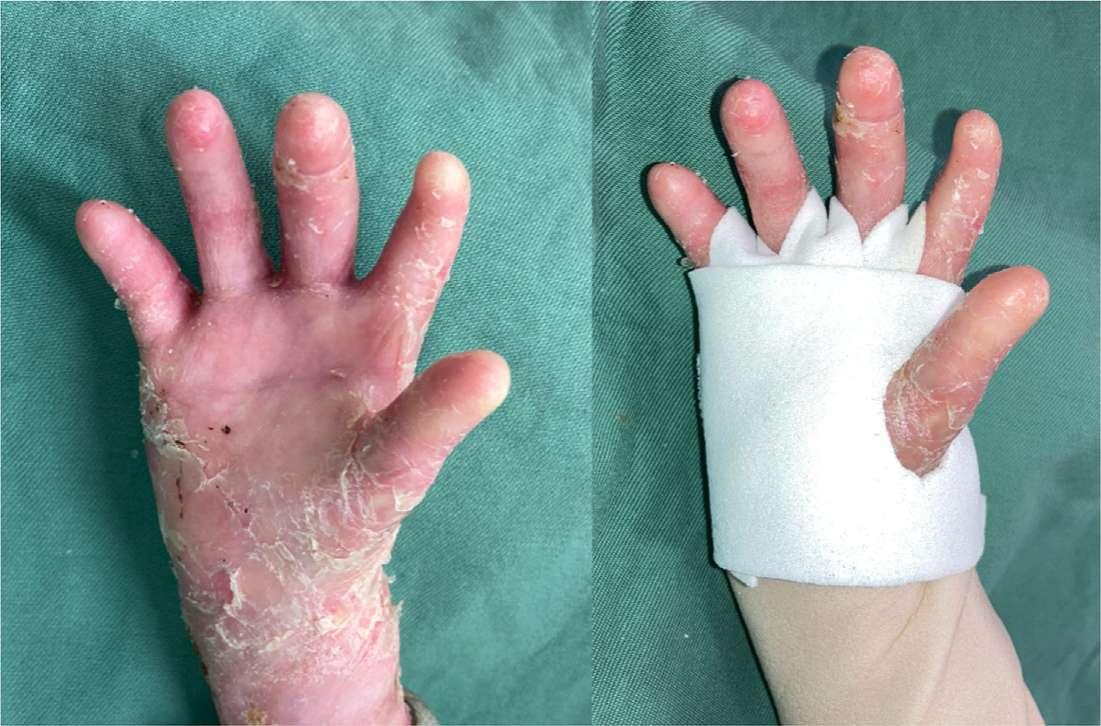
Digital separation was maintained at 6 months postoperatively (left). Mepilex Lite® was applied longitudinally across the interdigital spaces extending to the wrist (right).

The patient was discharged on the day after surgery. On postoperative day 4, outpatient dressing removal confirmed engraftment of the cultured epidermis. Mepilex Lite® (Mölnlycke Health Care, Gothenburg, Sweden) was then applied to all web spaces, with sufficient length to extend beyond the wrist. The same dressing was wrapped circumferentially around the entire hand to secure fixation of all web spaces (Figure 4). No additional bandage fixation was used in order to minimize pain.

This care protocol was continued three times per week, primarily at home. Complete epithelialization was achieved within 3 weeks, after which rehabilitation was initiated.

At 2 months postoperatively, a marked improvement in range of motion was observed. The %TAM improved from 7.7% to 46.2% in the index finger and from 3.9% to 65.4% in the middle through small fingers. At 6 months, no recurrent contracture was observed, and the improved range of motion was maintained.

## Discussion

Long-term improvement or complete prevention of recurrence of pseudosyndactyly in RDEB is still challenging, although digital separation has been developed for decades.^1^, ^2^, ^3^, ^4^, ^5^ Simple incision alone has been reported to result in recurrence in >50% of cases within 1 year.^1^ A report from the 1970s using split-thickness skin grafts (STSG) described recurrence within 1 year in all 10 treated hands.^2^ Full-thickness skin grafts (FTSG) are widely applied to cover skin defect; however, it has been reported that recurrence rates are greater than 50% within 1.5 years.^3^ A hybrid approach using both FTSG and artificial dermal dressing has shown promising short-term outcomes, with recurrence as low as 3% at 1 year, yet long-term follow-up, over 6 years, has revealed 77% of recurrence rate.^4^ These suggest that long-term complete prevention of recurrence in EB-related pseudosyndactyly remains challenging. Therefore, instead of pursuing complete prevention of recurrence, prioritizing a minimal-invasiveness approach is considered to be one of options that can be safely repeated when recurrence occurs.^4^, ^5^, ^6^

In EB scars, the thickened dermis is considered a major cause of contracture. Previous study reported that tenascin-C–positive collagen is prominent in the superficial dermis layer, whereas its signal diminishes in the deeper layer,^7^ indicating that the characteristic of fibrosis differs between the superficial and deep dermis. Intraoperatively in this case, contracture was progressively released as the superficial dermis was incised, and the deeper dermal layers were confirmed to be softer (Movie 1). Microsurgical technique enabled real-time identification of the border between the firm dermal layer and soft dermal layer, which allowed preserve non-contractive tissue selectively.

One limitation of this study is the short follow-up period. Although long-term recurrence may occur, our strategy prioritizes repeatability. Minimizing the physical and psychological burden on patients and their families represents a meaningful clinical advantage, particularly when re-intervention becomes necessary.

## Conclusions

Considering the potential recurrence in EB-related pseudosyndactyly, our concept prioritized repeatability even when contracture recurs. A minimally invasive microsurgical technique allowed selective release of the scarred tissue, achieving short-term functional improvement without recurrence. This approach may represent a conceptual shift from recurrence prevention to recurrence-tolerant management in RDEB hand reconstruction.

## Declaration of AI and AI-assisted technologies in the writing process

During the preparation of this manuscript, the authors used ChatGPT (OpenAI) to improve the readability and language of the text. After using this tool, the authors reviewed and edited the content as needed and take full responsibility for the content of the published article.

## Declaration of competing interest

The authors have no financial interest to declare.

## Appendix Supplementary materials

Image, video 1 dummy alt text
